# Measurement Technologies for Ankle-Dorsiflexion Function After Stroke: A Systematic Review and Meta-Analysis of Sensing Approaches and Their Relationships with Gait Performance

**DOI:** 10.3390/s26113598

**Published:** 2026-06-05

**Authors:** Hiroki Ito, Hideaki Yamaguchi, Ryosuke Yamauchi, Ken Kitai, Takayuki Kodama

**Affiliations:** 1 Graduate School of Health Science, Kyoto Tachibana University, Yamashina-ku, Kyoto 607-8175, Kyoto Prefecture, Japan; h901524007@st.tachibana-u.ac.jp (H.I.); caretech.plus.hy@gmail.com (H.Y.); h901522007@st.tachibana-u.ac.jp (R.Y.); h901523004@st.tachibana-u.ac.jp (K.K.); 2 Comprehensive Rehabilitation Center, Ōmi Onsen Hospital, Kōjinkai Medical Corporation, Higashiomi 527-0145, Shiga, Japan; 3 CARETECH plus, Nagoya 462-0847, Aichi, Japan; 4 Cognitive and Molecular Research Institute of Brain Diseases, Kurume University, Kurume 830-0011, Fukuoka, Japan

**Keywords:** stroke rehabilitation, ankle dorsiflexion, gait performance, sensing technology, handheld dynamometry

## Abstract

**Highlights:**

**What are the main findings?**

- Sensor-based assessments after stroke mainly use handheld dynamometers, load cells, or isokinetic dynamometers. Ankle-dorsiflexor strength generally correlates positively with gait speed and endurance.
- Across studies, the strength of the association between ankle-dorsiflexion indices and walking speed varied widely depending on the sensing technology, dorsiflexion metric, and patient characteristics. Quantitative pooling was therefore treated as exploratory and used only to complement the qualitative synthesis of these heterogeneous patterns.

**What are the implications of the main findings?**

- Handheld dynamometry is a practical and reasonably valid tool for the routine assessment of ankle-dorsiflexor strength in stroke rehabilitation.
- Standardized dorsiflexion protocols and scalable sensor systems are required to better predict gait outcomes and guide ankle-focused interventions.

**Abstract:**

Ankle dorsiflexion plays a vital role in ensuring safe and effective walking post-stroke, yet the best methods for assessing it and their clinical significance are still uncertain. This research compiles the existing sensor-based technologies used to measure ankle dorsiflexion in adults who have experienced a stroke and examines how these measurements correlate with walking performance. It also compares these findings with traditional clinical evaluation methods like manual muscle testing (MMT). We conducted a systematic search of PubMed, IEEE Xplore, and the Cochrane Library (2000–2025) for both observational and experimental studies that utilized sensor-based techniques (such as handheld or isokinetic dynamometry, load cells, and proprioceptive devices) to quantify ankle dorsiflexion and reported their relationship with gait outcomes. Additionally, studies employing conventional, non-instrumented clinical grading (e.g., ankle-dorsiflexor MMT) were included if they explored the connection between ankle function and gait, although these were not included in the quantitative analysis. Eighteen studies involving 783 stroke survivors met the inclusion criteria and were evaluated using the Newcastle–Ottawa Scale. Generally, individual studies found a positive association between ankle-dorsiflexor strength and both gait speed and endurance, although some negative correlations were noted. The strength and sometimes direction of these associations varied depending on the sensing technology, dorsiflexion index, gait outcome, and stroke chronicity. Overall, the current evidence indicates a generally positive but highly variable relationship between ankle dorsiflexion measurements and gait post-stroke, emphasizing the need to identify sources of variability and to create standardized, clinically applicable sensor-based assessment protocols.

## 1. Introduction

Stroke is a leading cause of long-term disability worldwide, and gait impairment is one of the most functionally limiting consequences for survivors [1]. Approximately 80% of patients with stroke experience some degree of walking dysfunction, and the restoration of independent ambulation is consistently ranked as a top priority by patients undergoing rehabilitation [2,3]. The ability to walk safely and efficiently is fundamental to maintaining independence in activities of daily living, community participation, and the overall quality of life.

Gait after stroke is characterized by reduced speed, asymmetry, increased energy expenditure, and heightened fall risk [4,5]. The pathophysiology of poststroke gait dysfunction is multifactorial and involves weakness, spasticity, impaired proprioception, and altered motor planning; therefore, the relative contributions of different lower-limb segments to overall gait impairment have been the subject of considerable research [6,7]. Accumulating evidence suggests that distal lower-limb function, particularly at the ankle, may play a more critical role in determining functional gait outcomes than previously considered [8,9].

Ankle dorsiflexion is essential for normal gait mechanics. During the swing phase, adequate dorsiflexion ensures foot clearance and prevents tripping; whereas, during the stance phase, controlled eccentric dorsiflexion contributes to shock absorption and forward progression [10]. To achieve these functions, the tibialis anterior muscle, which is the primary ankle dorsiflexor, must be precisely activated with the appropriate timing and magnitude. Impaired ankle dorsiflexion after stroke manifests clinically as foot drop, a condition characterized by an inability to lift the forefoot during the swing phase, leading to compensatory strategies such as hip hiking, circumduction, or vaulting on the unaffected limb [11]. These compensations increase energy expenditure and contribute to the characteristic inefficiency of the hemiplegic gait. Even among individuals who regain the ability to walk, residual deficits in ankle control are common, and many stroke survivors continue to rely on ankle–foot orthoses to secure foot clearance and stability during gait [12]. These observations highlight the importance of adequately assessing ankle-dorsiflexion function to understand and manage gait disturbances after stroke.

Prognostic prediction plays a central role in treatment planning and goal setting for stroke rehabilitation [13]. Clinicians must identify, as early as possible, both future walking capability and current problems in ankle control that may limit gait [14]. Previous studies have explored the prediction of gait ability based on performance in other motor tasks, such as the sit-to-stand movement or static standing balance, as assessed using clinical scales and performance tests [15,16]. While these surrogate tasks can offer useful information, they have inherent limitations as prognostic indicators of gait: they differ biomechanically and neurologically from walking itself and may not adequately capture gait-specific motor control for the robust prediction of walking outcomes. Moreover, in patients with severe motor deficits, evaluations that require standing or walking often cannot be performed, further restricting their utility for early prognostication.

These limitations point to the need for assessment methods that can be applied even in the very early phase after stroke and in patients with more severe impairments, while specifically targeting ankle movements that are directly relevant to gait. From a practical standpoint, assessments that can be performed in resting or simple postural conditions (e.g., sitting or supine) without requiring the patient to stand or walk are highly desirable. Conceptually, even under these conditions, appropriately designed tasks and measurement systems may provide access not only to a simple joint range-of-motion or maximal muscle strength, but also to more complex aspects of gait-related ankle control. The quantitative evaluation of ankle-dorsiflexion performance under resting or minimally demanding conditions is a promising approach for deriving clinically valuable information regarding future gait performance.

Recent advances in sensing and measurement technologies provide new opportunities to quantify ankle-dorsiflexion control in detail [17]. Various sensor systems, including wearable inertial measurement units (IMUs), force and pressure sensors, robotic or mechatronic devices, surface electromyography (sEMG), motion-capture systems, and combined multimodal setups, have been proposed to capture the kinematic, kinetic, and neuromuscular aspects of ankle function [18,19]. Furthermore, experimental paradigms incorporate not only simple voluntary movements, but also rhythmic tasks, rapid goal-directed dorsiflexion, and motor imagery-based tasks [20,21]. However, despite the technological progress, several important questions remain insufficiently addressed from both the clinical and engineering perspectives:

(i) the characteristics of ankle dorsiflexion performance assessed at rest or in simple postures that are associated with gait ability or performance after stroke;

(ii) the types of sensor configurations, signals, and features that are most informative for capturing ankle “control” rather than mere capacity.

To the best of our knowledge, no systematic review has comprehensively synthesized the relationship between ankle-dorsiflexion control assessed under resting or simplified conditions and actual gait performance after stroke. Moreover, the literature has not characterized the advantages and limitations of the sensor systems and experimental paradigms used for such assessments. This lack of synthesis hinders the formulation of clear design requirements for future sensor-based evaluation systems and limits the translation of existing technologies into clinically meaningful tools for gait prognosis. Therefore, the primary aim of this systematic review was to qualitatively synthesize how different sensor-based approaches to assessing ankle-dorsiflexion function after stroke relate to gait performance and to identify methodological and clinical sources of heterogeneity across studies. A secondary aim was to place these sensor-based findings in the context of conventional clinical assessments such as manual muscle testing (MMT), when such measures were used to characterize the ankle–gait relationship. Quantitative meta-analysis of reported correlations was planned as an exploratory, secondary approach and was restricted to instrumented, sensor-based assessments to illustrate overall trends, rather than to derive a single definitive pooled effect size. Quantitative meta-analysis of reported correlations was planned as an exploratory, secondary approach to illustrate overall trends, rather than to derive a single definitive pooled effect size.

## 2. Materials and Methods

### 2.1. Protocol and Registration

This systematic review was conducted in accordance with the Preferred Reporting Items for Systematic Reviews and Meta-Analyses (PRISMA) 2020 guidelines [22]. The review protocol was developed a priori, specifying the research questions, eligibility criteria, search strategy, study-selection methods, data-extraction methods, and quality assessment. This review was not prospectively registered on PROSPERO. This protocol is available from the corresponding author upon reasonable request.

### 2.2. Search Strategy

A comprehensive literature search was conducted across three electronic databases: PubMed, IEEE Xplore, and the Cochrane Library. The search was performed on 22 January 2026 and covered publications from January 2000 to December 2025. Search strings combined four main concepts: (i) stroke population (e.g., “stroke”, “hemiplegia”, “cerebrovascular accident”); (ii) ankle dorsiflexion function (e.g., “ankle”, “dorsiflexion”, “tibialis anterior”); (iii) strength or central measures (e.g., “strength”, “torque”, “dynamometer”, “MVC”, “EEG”); and (iv) gait outcomes or prognostic relationships (e.g., “gait speed”, “walking capacity”, “6-min walk”, “Timed Up and Go”, “correlation”, “prediction”).

For PubMed, filters were applied to restrict results to human studies, English language, and the specified publication period. For IEEE Xplore, we limited records to conference and journal articles published between 2000 and 2025. In the Cochrane Library, the search was restricted to the same time frame. The exact database-specific search strategies are provided in Supplementary Table S1. In line with our clinical focus, the search strings were primarily constructed from conventional clinical terminology and dynamometry-related terms; therefore, proof-of-concept engineering studies describing novel wearable or flexible sensors without explicit clinical gait outcomes may not have been captured.

### 2.3. Eligibility Criteria

Studies were included if they met the following criteria. The study population consisted of adult patients (aged 18 years or older) with a clinical diagnosis of stroke (ischemic or hemorrhagic), regardless of the time since onset. Studies could be observational (cross-sectional, cohort, or case–control) or experimental in design provided that they reported the assessment of ankle-dorsiflexion parameters and their association with gait outcomes.

The assessment methods of interest included dynamometry-based strength measurement (isometric or isokinetic); motion-capture systems (3D optical, 2D video, or inertial sensor-based) for kinematic assessment; surface EMG for tibialis anterior muscle-activation assessment; and EEG-based methods, including corticomuscular coherence (CMC) and movement-related cortical potentials. The gait outcomes of interest included spatiotemporal parameters (such as gait speed, cadence, stride length, and symmetry indices), functional walking measures (such as the 6-Minute Walk Test and TUG), and measures of gait quality (such as the Gait Deviation Index and energy cost). Studies reporting the psychometric properties of the ankle-assessment methods (test–retest reliability, inter-rater reliability, concurrent validity, and predictive validity) were also included. Studies were required to have been published in English in peer-reviewed journals.

Studies were excluded if they focused exclusively on pediatric populations or nonstroke neurological conditions; if they examined only the effects of interventions without reporting baseline assessment data or associations; conference abstracts, dissertations, or review articles without original data; or if the full text was not available. In line with the focus on sensor-based assessments, we primarily sought studies that employed instrumented measurement technologies (e.g., dynamometry, load cells, robotic/proprioceptive devices, or combined load-cell and EMG setups) to quantify ankle-dorsiflexion function. However, studies that assessed ankle dorsiflexion solely using conventional, non-instrumented clinical grading scales such as manual muscle testing (MMT) were also retained when they reported an explicit association between ankle function and gait performance. These MMT-only studies were used to contextualize the ankle–gait relationship from the perspective of traditional clinical practice, but were not included in the quantitative meta-analysis.

### 2.4. Study Selection

The study selection was conducted in two phases. In the first phase, two reviewers independently screened the titles and abstracts of all identified records against the eligibility criteria. Records were classified as “include,” “exclude,” or “uncertain.” In the second phase, the full texts of potentially eligible studies were retrieved and independently assessed by both reviewers. If a consensus could not be reached, disagreements were resolved through discussion with a third reviewer.

### 2.5. Data Extraction

Data were extracted using a standardized form that captured study characteristics (authors, year, and study design), participant characteristics (sample size, age, sex, stroke type, and time since stroke), assessment technology and protocol details, gait-outcome measures, statistical methods for analyzing associations, and the main findings regarding the relationship between ankle-function measures and gait performance. Studies reporting correlation coefficients, regression coefficients, or other effect sizes were extracted, along with confidence intervals and *p*-values, where available. The data were extracted by a single reviewer and verified by a second reviewer.

### 2.6. Quality Assessment

The methodological quality of the included studies was assessed using the Newcastle–Ottawa Scale for observational studies [23]. This scale evaluates studies across three domains: selection of study groups (maximum of 4 stars), comparability of groups (maximum of 2 stars), and ascertainment of outcomes (maximum of 3 stars). Studies were rated as high-quality if they scored 7–9 stars, moderate-quality if they scored 4–6 stars, and low-quality if they scored 0–3 stars. Quality assessment was performed independently by two reviewers, and disagreements were resolved through discussion. Detailed quality-assessment results for all the included studies are provided in Table S1. Most of the included studies had a cross-sectional design. In line with common adaptations of the NOS for cross-sectional studies, items that are conceptually inapplicable to cross-sectional designs (e.g., S4: outcome not present at the start of the study; O2–O3: adequacy of follow-up) were marked as “not applicable” and were not penalised when deriving total quality scores.

### 2.7. Data Synthesis

We first performed a qualitative synthesis to describe patterns in the reported associations between ankle-dorsiflexion measures and gait performance. In addition, we conducted an exploratory meta-analysis to summarize these associations quantitatively, with the understanding that substantial clinical and methodological heterogeneity would limit the interpretability of any single pooled effect size. For each eligible study, the Pearson’s correlation coefficient r between the predictor (e.g., ankle-dorsiflexor strength) and gait speed was extracted or derived. When correlation coefficients were not reported directly, they were calculated from other statistics (e.g., regression coefficients with standard errors, t-values, or p-values) whenever possible, using standard formulas.

Because raw correlation coefficients were not normally distributed, we applied Fisher’s z transformation to each study-specific correlation coefficient, r:

$$z=\frac{1}{2}ln\left(\frac{1+r}{1-r}\right),Var(z)=\frac{1}{n-3},$$

where n is the sample size of the study.

We pooled study-specific Fisher’s z values using an inverse-variance weighted random-effects model, implemented with the DerSimonian–Laird method to estimate the between-study variance $\tau^{2}$. The pooled Fisher’s z estimates were then back-transformed to correlation coefficients r for interpretation and presented with their 95% confidence intervals (95% CIs).

Between-study heterogeneity was assessed using Cochran’s Q statistic and quantified using the $I^{2}$ statistic, which describes the percentage of total variation across studies due to heterogeneity rather than chance. As a rule, $I^{2}$ values of approximately 25%, 50%, and 75% represented low, moderate, and high heterogeneity, respectively. The estimated between-study variance from the random-effects model is reported as $\tau^{2}$.

The primary analysis combined all relevant predictors (ankle-dorsiflexor strength and central measures, where available) into an “All” group and examined their correlation with gait speed. In a predefined subgroup analysis, we restricted the dataset to studies that reported ankle-dorsiflexor strength as a predictor (“Strength” group) and conducted a separate meta-analysis using the same procedures. When multiple gait outcomes were reported in the same study (e.g., comfortable vs. maximal gait speed), we used a priori decision rules to select a single outcome per study (e.g., comfortable gait speed was prioritized; if not available, maximal gait speed was used). When multiple correlations were available for different conditions within a study (e.g., affected vs. unaffected limb, different test positions), we selected the condition judged a priori to be the most clinically relevant (e.g., affected limb, primary test condition) to avoid the double counting of the participants.

Of the 18 studies included in the qualitative synthesis, 17 studies that used instrumented, sensor-based assessments contributed at least one ankle-dorsiflexion index and a corresponding walking-speed outcome to the “All” group meta-analysis. Of these 17 sensor-based studies, 13 reported a directly quantified dorsiflexor strength variable (e.g., isometric torque, MVC force, peak torque) that could be extracted as a single predictor. These 13 studies formed the “Strength” subgroup meta-analysis. One included study assessed ankle dorsiflexion exclusively using MMT without instrumentation; this study was therefore retained only for the qualitative synthesis and was not entered into any meta-analytic pooling. The remaining five studies either focused primarily on proprioceptive or central measures, combined strength and non-strength indices in a way that precluded separate extraction, or did not report sufficient data for inclusion in the strength-only analysis, and were therefore retained only in the “All” group meta-analysis.

Studies that did not report a correlation coefficient or sufficient data to derive one were included in the qualitative synthesis, but were excluded from the quantitative meta-analysis. To explore the robustness of the findings and influence of potentially outlying studies (e.g., very small sample sizes or extremely large correlation coefficients close to ∣r∣=1), we conducted sensitivity analyses by temporarily excluding such studies and re-estimating the pooled effect size and heterogeneity indices ($I^{2}$, $\tau^{2}$), where applicable.

All statistical analyses were performed using Microsoft Excel (Microsoft Corp., Redmond, WA, USA) with customized calculation sheets. Two-sided tests were used throughout the study, with a significance level of p<0.05.

Given the anticipated heterogeneity in patient characteristics, sensing technologies, and outcome definitions, all quantitative pooling was considered exploratory and hypothesis-generating. Accordingly, the pooled correlation coefficients were interpreted only in conjunction with the qualitative synthesis and were not regarded as definitive estimates of the “true” strength of association.

## 3. Results

### 3.1. Study Selection

**Figure 1:**
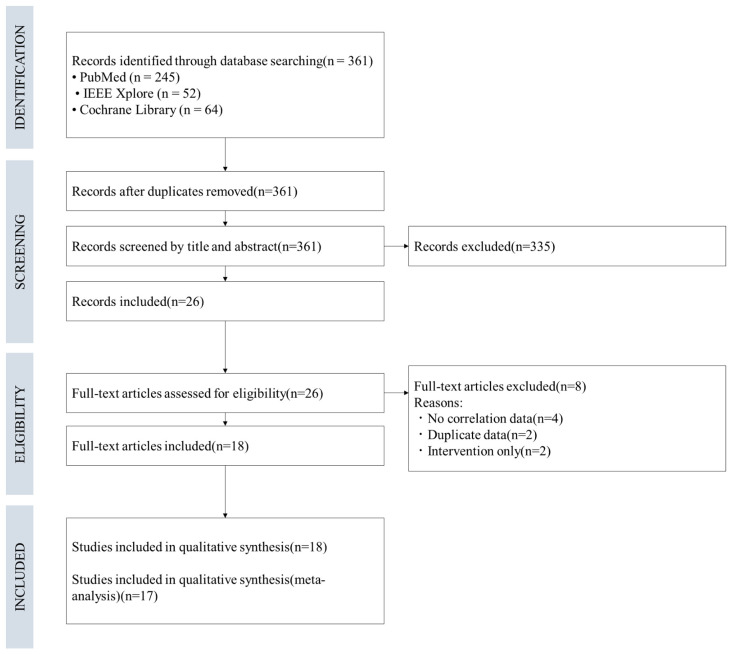
PRISMA 2020 flow diagram of study selection. A total of 361 records were identified through database searching (PubMed, IEEE Xplore, and Cochrane Library). After the removal of duplicates, all 361 records were screened by title and abstract, and 335 were excluded. Twenty-six records were retained, and 26 full-text articles were assessed for eligibility. Eight full-text articles were excluded (no correlation data, n = 4; duplicate data, n = 2; intervention only, n = 2). Eighteen studies were finally included in the qualitative synthesis, and 17 sensor-based studies were entered into the quantitative meta-analysis (Table S2) [24,25,26,27,28,29,30,31,32,33,34,35,36,37,38,39,40,41]. One study that assessed ankle dorsiflexion solely using non-instrumented MMT was excluded from the meta-analytic pooling but retained for qualitative comparison.

The systematic database search identified 361 potentially relevant studies. During the first screening stage based on titles and abstracts, 26 articles (7.2%) were classified as “include,” while 335 articles (93.0%) were classified as “exclude.” In the second stage, a full-text assessment was conducted for 26 articles; ultimately, 18 articles met the inclusion criteria and were included in this systematic review (Figure 1).

### 3.2. Study Characteristics

The characteristics of the 18 included studies are summarized in Table 1. The studies were published between 2003 and 2025 and represented over two decades of research on ankle-dorsiflexion assessment in patients with stroke. The sample sizes ranged from 7 to 105 participants, with 783 patients with stroke across all studies. The mean age of participants ranged from 54.6 to 69.5 years, and the majority of studies included more male than female participants. The time since stroke varied considerably across studies, ranging from the acute/subacute phases (35 days) to the chronic stages (up to 19 months or several years post-stroke).

Regarding the assessment devices, handheld dynamometers (HHD) were the most commonly used instruments, and they were employed in seven studies [24,25,26,27,28,29,30]. Load cells or force transducers were used in four studies [31,32,33,34], whereas isokinetic dynamometers (Biodex System, Kim-Com) were used in two studies [35,36]. One study used a custom-made dynamometer [37], another used an ankle-measuring proprioceptive device [38], another used a linear servomotor for movement-sense assessment [39], and another combined load-cell measurements with EMG [40]. While the primary focus of this review was on instrumented, sensor-based technologies, we also retained one study [41] that utilized manual muscle testing (MMT) without instrumentation as a conventional clinical comparator for the ankle–gait relationship; this MMT-only study was not entered into the quantitative meta-analysis.

The measured dorsiflexion indices included isometric strength (Nm/kg, N, kg), MVC force or torque, peak torque (Nm), residual muscle deficits (expressed as percentages), joint-position reproduction error for proprioceptive assessment, movement-sense scores, range of motion, and MMT grades. Walking-ability indices included gait velocity or walking speed (measured in m/s or cm/s), 6-min walk test (6 MWT) distance, TUG test time, and functional-ambulation categories.

### 3.3. Quality Assessment

Table S3 displays the methodological quality of the 18 studies included, evaluated using the Newcastle−Ottawa Scale. According to the adapted NOS, which has a maximum score of 6★, all studies achieved scores between 5 and 6★, indicating high methodological quality. In accordance with the adapted NOS, items S4, O2, and O3 were deemed “not applicable” for these studies and were thus excluded from the total score calculation. As a result, star ratings are provided solely for S1–S3, C1–C2, and O1. None of the studies were classified as low-quality.

In the selection domain, all 18 studies received stars for representativeness of the exposed cohort (S1), selection of the nonexposed cohort (S2), and ascertainment of exposure (S3).

For comparability, 18 studies (100%) controlled for age or severity (C1), whereas 11 studies (61.1%) controlled for additional confounding factors (C2). In the outcome domain, all studies received stars for the assessment of outcome (O1).

### 3.4. Correlation Between Ankle-Dorsiflexion Measures and Gait Performance

#### 3.4.1. Individual Study Results

The correlation coefficients between the ankle-dorsiflexion and walking ability indices in each study are presented in Table 1.

The correlations varied substantially across studies, ranging from strongly negative (r = −0.75) to strongly positive (r = 0.79).

Among studies measuring ankle-dorsiflexor strength directly, positive correlations with gait speed were reported by Mentiplay et al. (r = 0.67) [24], Lodha et al. (r = 0.34) [31], Kowal et al. (r = 0.65) [35], Dorsch et al. (r = 0.50) [27], Ng and Hui-Chan (r = 0.79) [33], Klein et al. (r = 0.75) [37], Kim et al. (r = 0.33) [36], and Kwong et al. (r = 0.41 with 6 MWT) [29]. Aguiar et al. reported a moderate positive correlation (r = 0.37) between ankle strength and a comfortable gait speed [30].

Conversely, several studies have reported negative correlations. Chan et al. [25] found a negative correlation (r = −0.39) between ankle-dorsiflexor strength and TUG motor time, which is consistent with a positive relationship between strength and functional mobility because lower TUG times indicate better performance. Similarly, Ng and Hui-Chan [34] reported a negative correlation (r = −0.67) between peak torque and TUG time. Ozgozen et al. [26] reported a strong negative correlation (r = −0.75) between residual muscle deficits (expressed as a percentage of nonparetic strength) and gait speed, indicating that greater residual deficits are associated with slower walking speeds. Chisholm et al. [32] found a negative correlation (r = −0.32) between the isometric MVC and swing phase peak dorsiflexion.

For studies assessing central or proprioceptive measures, Johnson et al. [38] reported a negative correlation (r = −0.28) between joint-position reproduction dynamic error and walking speed, suggesting that greater proprioceptive errors were associated with slower gait. Ng et al. [28] found a negative correlation (r = −0.48) between ankle strength and walking speed, which appears counterintuitive and may reflect methodological factors or sample characteristics. Lee et al. [39] reported a moderate positive correlation (r = 0.48) between movement sense and the 6 MWT distance. Cho et al. [41] found a moderately positive correlation (r = 0.56) between the MMT grade and walking level. Here, the results are calculated using Spearman’s correlation, based on MMT grades ranging from 0 to 5 and a proprietary gait level index.

Negro F et al. [40] used a combined load cell and EMG assessment and reported a correlation coefficient derived from R^2^ = 0.51 (r = 0.71) between MVC and task-duration measures with walking speed.

#### 3.4.2. Meta-Analysis Results

**Figure 2:**
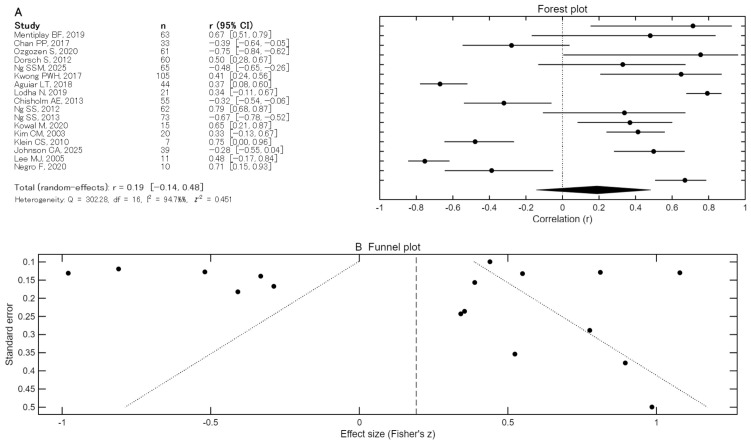
(**A**) Forest plot [24,25,26,27,28,29,30,31,32,33,34,35,36,37,38,39,40] and (**B**) funnel plot for the meta-analysis of correlations between all ankle-dorsiflexion indices (“All” group; including strength, proprioceptive, and central measures) and walking speed in patients with stroke. In the forest plot, individual studies are shown with correlation coefficients and 95% confidence intervals; the vertical dotted line indicates the pooled correlation estimated with a random-effects model, and the black diamond represents the pooled random-effects estimate with its 95% confidence interval. In the funnel plot, each dot represents an individual study, the vertical dashed line indicates the pooled effect size, and the diagonal dotted lines denote the pseudo-95% confidence limits around the pooled effect. The single included study using non-instrumented manual muscle testing (Cho KH 2014 [41]) was not entered into this analysis.

**Figure 3:**
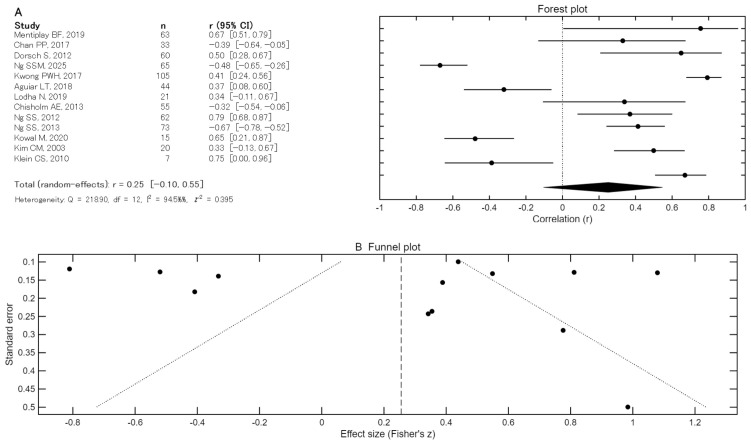
(**A**) Forest plot [24,25,27,28,29,30,31,32,33,34,35,36,37] and (**B**) funnel plot for the meta-analysis restricted to studies assessing ankle-dorsiflexor strength (“Strength” group) and walking speed in patients with stroke. Individual studies are shown with correlation coefficients and 95% confidence intervals, and the diamond represents the pooled random-effects estimate under the random-effects model. Vertical dashed lines indicate the null effect and the 95% confidence limits in the funnel plot.

Because of the wide variability in study designs, sensing technologies, and gait outcomes, the meta-analysis was prespecified as an exploratory complement to the qualitative synthesis. The results of this exploratory pooling are summarized in Table 2 and Figure 2 and Figure 3.

Seventeen of the 18 included studies, all of which used instrumented, sensor-based assessments, contributed at least one ankle-dorsiflexion index and a corresponding walking-speed outcome to the “All” group analysis. The remaining study (Cho KH 2014 [41]), which assessed ankle dorsiflexion using non-instrumented MMT only, was not entered into the meta-analysis. As noted in the Methods, a subset of 13 studies that reported a clearly defined dorsiflexor strength measure (e.g., isometric torque, MVC force) were additionally entered into the “Strength” subgroup analysis, whereas studies focusing primarily on proprioceptive or central indices, or those with insufficient strength data, were not included in this subgroup.

For the exploratory analysis including all 18 studies with both strength and central measures (“All” group), the pooled correlation coefficient between ankle-dorsiflexion indices and walking speed was r = 0.21 (95% CI: −0.11 to 0.49) using a random-effects model. This correlation was not statistically significant, as the 95% confidence interval crossed zero. Substantial heterogeneity was observed, with I^2^ = 94.56% and τ^2^ = 0.44, indicating that approximately 95% of the observed variance in correlation coefficients was attributable to between-study heterogeneity rather than sampling error. In light of this, the pooled value is best viewed as a descriptive summary rather than a precise estimate of a single underlying effect.

In the subgroup analysis restricted to 13 studies that specifically measured ankle-dorsiflexor strength (“Strength” group), the pooled correlation coefficient was r = 0.65 (95% CI: −0.42 to 0.96) (Figure 3).

While this point estimate suggests a moderate-to-strong positive correlation, the wide confidence interval crossing zero indicates that the association was not statistically significant. Heterogeneity remained high in this subgroup, with I^2^ = 94.52% and τ^2^ = 0.40. Thus, this subgroup meta-analysis further illustrates the diversity of study-specific associations, rather than providing a robust common effect size.

Given that only 13 studies contributed to the strength subgroup, the power of the funnel plot to detect publication bias is limited, and the plot is presented for descriptive purposes only. The substantial heterogeneity observed in both analyses suggests that the relationship between ankle-dorsiflexion measures and gait performance is influenced by multiple moderating factors, including the specific measurement device used, the dorsiflexion index assessed, the gait-outcome measure employed, patient characteristics such as time since stroke and baseline functional status, and methodological differences across studies.

## 4. Discussion

### 4.1. Summary of Main Findings

This systematic review and meta-analysis examined the relationship between ankle-dorsiflexion function and gait performance in patients with stroke, with particular attention paid to the sensing technologies and measurement approaches used for assessment. Eighteen studies involving 783 patients with stroke were included, representing a comprehensive synthesis of the current literature on this topic.

Across the included studies, ankle-dorsiflexor strength was generally positively associated with gait speed, endurance, and functional mobility, whereas measures reflecting greater impairment (e.g., residual strength deficits, larger proprioceptive errors, longer TUG times) showed negative correlations with gait performance. The magnitude and even direction of these correlations varied according to the sensing technology employed, the specific dorsiflexion metric, the gait outcome selected, and the clinical profile of the sample. Exploratory meta-analytic pooling yielded small-to-moderate positive overall trends, but with very wide confidence intervals crossing zero and extremely high heterogeneity (I^2^ > 94%). These findings confirm that no single summary correlation can adequately describe the ankle–gait relationship across the diverse contexts represented in the literature. Consequently, in this Discussion we place greater emphasis on the qualitative patterns and potential sources of heterogeneity than on the pooled point estimates themselves.

Quality assessment using the Newcastle−Ottawa Scale indicated that most of the included studies (83.3%) were of high methodological quality, providing reasonable confidence in the validity of individual study findings. However, the marked heterogeneity across studies highlights the complexity of the relationship between ankle function and gait in patients with stroke. In addition to these sensor-based findings, one included study assessed ankle dorsiflexion using conventional MMT grading and reported a moderate positive association between MMT grade and walking level (Cho KH, 2014 [41]). This MMT-based evidence was used to contextualize the ankle–gait relationship from a traditional clinical perspective and was therefore summarized qualitatively but not included in the quantitative pooling.

### 4.2. Comparison with Previous Literature

The finding that ankle-dorsiflexor strength is positively associated with gait speed in patients with stroke is consistent with previous research emphasizing the importance of distal lower limb function in walking ability. Dorsch et al. [27] previously demonstrated that ankle-dorsiflexor strength significantly contributed to walking speed in independent ambulatory stroke survivors, a finding corroborated by the strong correlation observed in their study (r = 0.50). Similarly, Ng and Hui-Chan [33] have consistently reported moderate-to-strong correlations between ankle-muscle strength and walking endurance, as reflected in the r = 0.79 correlation with gait velocity reported in their 2012 study.

The negative correlations observed with TUG time in studies by Chan et al. [25] (r = −0.39) and Ng and Hui-Chan [34] (r = −0.67) are consistent with expectations, as lower TUG times indicate better functional mobility. When interpreted in terms of functional performance, these findings are consistent with the positive associations between strength and gait speed reported in other studies.

The heterogeneity in correlation magnitudes across studies may be partially explained by differences in patient populations. The relationship between ankle dorsiflexor strength and gait performance may differ between the acute and chronic stroke phases. Previous research has suggested that motor-recovery patterns stabilize at approximately 3−6 months post-stroke, after which, the relationship between muscle strength and functional outcomes may become more consistent [8,42]. The precision of the measurement instruments may have influenced the magnitude of the observed correlations. When examining the empirical data across studies, it is also apparent that sample size and study design modulate the observed strength of association. For example, two of the largest studies using hand-held dynamometry (Kwong et al., n = 105, r = 0.41 with 6MWT distance; Ng SSM et al., 2025 [28], n = 65, r = −0.48 with walking speed) reported only modest or even counterintuitive correlations, whereas smaller studies using load cells or isokinetic dynamometers often reported stronger positive associations (e.g., Ng SS 2012 [33], n = 62, r = 0.79; Kowal et al., 2020 [35], n = 15, r = 0.65). This pattern suggests that very high correlation coefficients in small samples should be interpreted with caution, and that differences in sampling and analytical choices likely contribute to the heterogeneity seen in the meta-analysis. Previous methodological studies demonstrated that isokinetic dynamometry and load cell-based systems provide higher measurement precision and reliability than manual muscle testing, which may contribute to a more accurate detection of true associations [43,44].

### 4.3. Assessment Technologies and Their Clinical Implications

The included studies employed diverse sensing technologies for ankle-dorsiflexion assessment, each with distinct advantages and limitations in clinical and research applications.

HHD emerged as the most commonly used assessment method, employed in 7 of the 18 studies. HHD offers practical advantages, including portability, relatively low cost, and ease of use in clinical settings. The studies using HHD demonstrated correlation coefficients ranging from 0.41 to 0.67 with gait outcomes, suggesting acceptable validity for detecting associations with walking performance. However, HHD measurements can be influenced by the examiner’s strength and technique, potentially introducing measurement error [43].

The load cells and force transducers used in four studies provided more standardized force measurements with reduced examiner dependency. Studies using these devices reported correlations ranging from 0.34 to 0.79, with higher values potentially reflecting greater measurement precision of these instruments. The disadvantage of load-cell systems is their typical requirement for fixed mounting configurations, which limits their portability and clinical feasibility [44,45].

From a broader clinical perspective, manual muscle testing (MMT) remains the most widely used bedside method for grading ankle-dorsiflexor weakness after stroke. In the present review, one study (Cho KH 2014 [41]) used MMT grades to characterize gait-related walking levels and found a moderate positive association. While this finding aligns qualitatively with the general notion that better dorsiflexor function supports higher walking ability, MMT is a non-instrumented, ordinal measure and lacks the measurement precision of sensor-based devices. For this reason, and to maintain methodological coherence, the MMT-only study was not entered into the quantitative meta-analysis and was instead used to contextualize sensor-based results against traditional clinical grading.

Isokinetic dynamometry, employed in two studies, represents the gold standard for muscle-strength assessment, allowing the measurement of force production throughout the range of motion at controlled velocities [44]. However, the high cost and space requirements of the isokinetic systems limit their application in most clinical settings.

The inclusion of studies assessing proprioceptive and central measures (Johnson et al., 2025 [38], Lee et al., 2005 [39]) expands the scope of ankle-function assessment beyond simple strength measurements. The moderate correlations observed between proprioceptive indices and gait outcomes (r = −0.28 for position sense error, r = 0.48 for movement sense) suggest that sensory aspects of ankle control also contribute to walking ability, though the evidence remains limited [46,47,48].

To fully appreciate the clinical utility of these assessment technologies, their inherent technical limitations and the biomechanical relevance of the extracted parameters must be considered. Although handheld and load-cell-based dynamometers are practically viable, their measurement fidelity is highly dependent on sensor fixation and alignment [44]. Off-axis loading and nonrigid fixation can introduce mechanical hysteresis and compromise the signal-to-noise ratio [49]. Moreover, minor deviations in the moment arm due to inconsistent sensor placement can significantly bias the derived torque values. This issue is particularly prevalent in HHD measurements, where the variable counterforce dynamics introduced by the examiner’s manual resistance can diminish the measurement reliability.

In systems incorporating multimodal approaches, such as surface electromyography (sEMG), technical challenges must be addressed rigorously. Variations in skin impedance, motion artifacts, and signal crosstalk from adjacent muscles (e.g., peroneal muscles or calf antagonists) require robust signal processing [50]. The extraction of reliable neuromuscular features requires high sampling rates (e.g., >1000 Hz) [51] and meticulous filtering algorithms to isolate the true neural drive to the tibialis anterior, which is particularly challenging in stroke survivors who frequently exhibit spasticity or abnormal co-contraction patterns [52,53].

From a biomechanical and neurophysiological perspective, exclusive reliance on the MVC may not fully encapsulate the complex motor control required for gait [54]. During walking, the ankle dorsiflexors must be activated rapidly and with precise timing, concentrically for foot clearance during the swing phase and eccentrically for shock absorption at heel strike [55]. Therefore, dynamic sensor features that capture the rate of force development, electromechanical delay, or dynamic proprioceptive error may provide a more functionally relevant proxy for walking ability than the absolute peak force. To advance stroke-rehabilitation diagnostics, future sensor-based systems should prioritize high-frequency data acquisition and standardized fixation protocols to capture these rapid temporal dynamics accurately.

### 4.4. Heterogeneity and Moderating Factors

The substantial heterogeneity observed in the present meta-analysis (I^2^ > 94%) warrants careful consideration. Several factors are likely to have contributed to this heterogeneity. Critically, while the pooled point estimates suggest positive trends (*r* = 0.21 for all measures and *r* = 0.65 for strength-only measures), both 95% confidence intervals cross zero, rendering the overarching pooled correlations statistically nonsignificant. This lack of statistical significance must be interpreted in the context of the exceptionally high heterogeneity (I^2^ > 94%), indicating that a singular summary effect size cannot reliably describe the complex relationship between ankle dorsiflexion and gait speed. The variability in sensor fidelity, ranging from manual handheld dynamometers to rigid floor-mounted load cells, is likely a substantial moderating factor. Differences in sensor resolution, sampling frequencies, and mechanical rigidity of the testing setups introduce varying degrees of measurement error, which directly dilute the strength of the observed clinical correlations. Furthermore, the temporal phase of stroke recovery (e.g., acute versus chronic) significantly alters the neuromuscular strategy during walking, indicating that the reliance on distal ankle control versus proximal compensatory mechanisms fluctuates widely across the evaluated cohorts. Consequently, the integrated evidence requires cautious interpretation, emphasizing that the validity of these functional predictions is highly contingent on the specific sensing architecture and clinical phase of the patient. In light of these considerations, we consider the pooled meta-analytic correlations to be hypothesis-generating only. A more informative approach is to focus on how the strength and direction of associations change across subgroups defined by sensing technology, dorsiflexion metric, gait outcome, and patient characteristics, that is, to qualitatively analyse the sources of heterogeneity rather than to rely on a single aggregated pooling point.

First, the diversity of measurement instruments and protocols across the studies introduces methodological heterogeneity. Different devices measure different aspects of muscle function (e.g., isometric vs. isokinetic strength and peak force vs. rate of force development), and variations in the testing positions, stabilization methods, and instruction sets can affect the measured values [56,57]. In our dataset, studies using more standardized, device-fixed measurements such as load cells or isokinetic dynamometers tended to report higher positive correlations with gait outcomes than some of the larger hand-held dynamometry studies, although this pattern was not uniform. This underscores that both instrument precision and how the test is implemented in practice can influence the magnitude and reliability of observed associations.

Second, heterogeneity in patient populations, including differences in the time since stroke, stroke severity, lesion location, and functional status, may influence the strength of the associations between ankle function and gait [48]. This relationship may differ between patients with acute and chronic stroke or between those with mild and severe motor impairments.

Third, the variety of gait-outcome measures employed (gait speed, 6MWT, and TUG) capture different aspects of walking performance [58,59]. Gait speed reflects instantaneous walking capacity, 6MWT incorporates endurance components, and TUG adds elements of transfers and turning. The strength of the correlation with ankle function may vary, depending on the aspect of the gait assessed.

Fourth, some studies reported negative correlations that appear counterintuitive (e.g., Ng et al. [28]: r = −0.48 between strength and walking speed). Such unexpected findings may arise from several sources, including ceiling or floor effects in the sample, confounding variables not controlled in the analysis, differences in measurement protocols, and statistical artifacts in small samples. Additionally, in heterogeneous stroke populations, compensatory strategies may obscure the direct relationship between single-joint strength and complex functional tasks, such as walking [60,61].

### 4.5. Clinical Implications

Despite the wide confidence intervals and substantial heterogeneity observed across studies, the generally positive pattern between ankle-dorsiflexor strength and gait performance underscores the critical clinical need for routine objective quantification of ankle function during stroke rehabilitation. While HHD currently provides a pragmatic balance between accessibility and baseline validity for everyday clinical use, its inherent susceptibility to examiner-induced variability limits its utility in developing robust, predictive prognostic models. Therefore, the primary clinical implication of this review is an important future direction to translate highly reliable, laboratory-grade sensing technologies, such as rigid-load cells or simplified mechatronic devices, into portable, user-friendly form factors that can be seamlessly integrated into standard clinical workflows without sacrificing measurement fidelity.

Beyond simple walking speed, the included studies related ankle dorsiflexion measures to endurance (6-Minute Walk Test), functional mobility (Timed Up and Go), and categorical walking levels. Correlations with 6MWT distance were generally moderate to strong (e.g., Ng SS 2012 [33], r = 0.79; Kwong PWH 2017 [29], r = 0.41), suggesting that dorsiflexor strength may be particularly relevant for sustaining repetitive stepping and community ambulation. In contrast, associations with TUG time were more variable, reflecting the influence of transfers, turning, and balance demands that are only partly determined by ankle function. These differences indicate that the clinical impact of dorsiflexion impairment may depend on which aspect of gait performance is considered instantaneous speed, endurance, or complex functional mobility.

Furthermore, the reviewed literature demonstrated that peripheral strength alone does not completely encapsulate the complex neurological deficits that impair gait. The inclusion of proprioceptive and central measures highlighted the equal importance of sensory feedback, coordination, and motor control. This finding strongly advocates for a clinical paradigm shift from unidimensional strength testing to comprehensive sensor-based neuromotor profiling. For clinicians, the future deployment of multimodal sensing setups capable of simultaneously capturing kinetic output, kinematic precision, and neuromuscular activation will be instrumental in accurately phenotyping stroke survivors. Implementing this high-resolution, sensor-driven assessment strategy may help rehabilitation professionals to move beyond generic strengthening protocols and design highly personalized, precise rehabilitation interventions tailored to the specific mechanical or neural bottlenecks of an individual patient’s paretic ankle.

### 4.6. Strengths and Limitations

This systematic review has several strengths. The comprehensive search strategy across multiple databases, use of established quality assessment tools, and quantitative synthesis through meta-analysis provide a rigorous evaluation of the available evidence. The inclusion of 18 studies with 783 patients represents a substantial evidence base, and the high methodological quality of most included studies supports the validity of the individual findings.

However, this study had several limitations. Substantial heterogeneity limits the interpretability of the pooled estimates and suggests that a single summary correlation may not adequately represent the true relationships across different populations and methodological contexts. The predominance of cross-sectional study designs limits causal inference. While correlations suggest associations, they do not establish that improving ankle strength will necessarily improve gait performance. Publication bias may have influenced the findings because studies reporting significant associations are more likely to be published than those with null results. An additional notable limitation pertains to the reliance of the search strategy on established clinical terminology and traditional dynamometric terms. By prioritizing clinically validated outcomes, the literature search may have inadvertently excluded purely engineering-focused proof-of-concept studies detailing novel wearable sensors such as IMUs, smart textiles, and unobtrusive flexible sensors. Consequently, the current synthesis predominantly reflects mature, commercially available clinical devices rather than cutting-edge ubiquitous sensing technologies. This limits the ability of this review to evaluate the predictive validity of next-generation unconstrained continuous-monitoring systems, which represent a rapidly expanding frontier in neurorehabilitation assessment. Finally, the restrictions on English-language publications may have excluded relevant studies published in other languages. Moreover, we did not have access to individual participant data, and key clinical variables such as detailed stroke severity, presence of contractures, and lesion characteristics were often reported inconsistently or not at all. As a result, we were unable to perform more advanced comparative analyses (e.g., meta-regression) or to define homogeneous subgroups based on clinical profile and measurement technology quality. Future individual participant data meta-analyses and large prospective cohorts using harmonized assessment protocols will be essential to clarify how these factors modify the relationship between ankle dorsiflexion and gait performance. Accordingly, while we reported exploratory pooled correlations for completeness, the most meaningful insights from this review arise from the qualitative comparison of subgroups and sensing approaches rather than from any single summary statistic. These recommendations are intended as hypothesis-generating suggestions based on the heterogeneous cross-sectional evidence summarised in this review and should not be interpreted as empirically proven effects.

### 4.7. Recommendations for Future Research

Based on the findings of this review, several recommendations for future research have been made. The standardization of measurement protocols for ankle-dorsiflexion assessment would facilitate comparisons across studies and reduce methodological heterogeneity. Longitudinal studies examining how changes in ankle function are related to changes in gait performance would strengthen the causal inferences. The investigation of moderating factors, including stroke chronicity, severity, and lesion location, would help identify the subgroups in which ankle function is most strongly related to gait outcomes. The development and validation of wearable sensor systems for continuous ankle-function monitoring can enhance the clinical assessment capabilities. Finally, randomized controlled trials specifically targeting ankle-dorsiflexor strengthening would provide direct evidence for the therapeutic value of this approach. Future research should focus on the integration of advanced, continuous, wearable sensing technologies to bridge the gap between resting ankle assessment and dynamic gait performance. Rather than relying exclusively on discrete laboratory-bound measurements using traditional dynamometry, future assessment paradigms should deploy multimodal wearable systems that combine miniature IMUs, flexible strain sensors, and wireless high-density sEMG. These unobtrusive systems would enable the continuous capture of ecological ankle kinematics and multimuscle activation patterns in free-living environments. Such an approach would facilitate the extraction of dynamic real-world control features, overcoming the ecological limitations of cross-sectional resting-state assessments. Furthermore, applying machine-learning algorithms to these high-dimensional continuous datasets may identify novel digital biomarkers of ankle function that are more sensitive and predictive of long-term gait recovery than isolated maximum voluntary contraction values. Developing highly scalable and clinically deployable sensor networks with standardized data-processing pipelines should be the primary objective for engineering and rehabilitation communities to move forward. In addition, collaborative efforts that share raw datasets across centres would enable individual participant data meta-analyses and more refined subgroup comparisons (e.g., by stroke severity, presence of contractures, or specific sensor technologies), thereby yielding more precise and clinically actionable estimates of the ankle–gait relationship.

## 5. Conclusions

This systematic review and meta-analysis examined the relationship between ankle-dorsiflexion function and gait performance in patients with stroke across 18 studies involving 783 participants.

Taken together, the available evidence indicates a generally positive, but highly variable, association between ankle-dorsiflexor strength and gait performance in patients with stroke. This relationship cannot be captured adequately by a single pooled correlation because it depends strongly on the sensing technology, specific dorsiflexion metric, gait outcome, and clinical profile of the population studied. HHD is the most commonly employed assessment technology, offering practical advantages for clinical implementation despite some limitations in measurement precision. The high heterogeneity observed underscores the need for standardized ankle-function assessment protocols and for future studies that explicitly examine how methodological and patient-level factors modify the ankle–gait relationship. Clinicians should therefore consider ankle-dorsiflexor strength as one component within a broader, multimodal assessment of gait, while recognizing that its prognostic value will differ across subgroups and measurement contexts.

## Figures and Tables

**Table 1:** Characteristics of included studies and correlation coefficients.

Study	Year	Sample Size	Sex (Male/Female), N	Age (Years)	Paretic Side (Left/Right), N	Time Since Stroke	Device	Dorsiflexion Index	Value	Walking-Ability Index	Value	Correlation Coefficients
Mentiplay BF [24]	2019	63	34/29	60 ± 13	33/30	39 ± 51 (months)	HHD (Lafayette Instrument Company, Lafayette, IN, USA)	Isometric strength (Nm/kg)	0.13 ± 0.09 (Nm/kg)	Walking speed (m/s)	Habitual pace 0.85 ± 0.37 (m/s) Fast pace 1.07 ± 0.47 (m/s)	0.67
Chan PP [25]	2017	33	22/11	60.2 ± 6.4	9/24	9.35 ± 4.28 (years)	HHD (Lafayette Instrument Company, Lafayette, IN, USA)	Strength (kg)	9.49 ± 5.47 (kg)	TUG motor time (s)	18.31 ± 5.77 (s)	−0.39
Ozgozen S [26]	2020	61	36/25	54.6 ± 11.7	31/30	19 (months)	HHD (Hoggan Health Industries Inc., West Jordan, UT, USA)	Residual deficits of the muscle groups (%): 100 − [(paretic muscle strength/non-paretic muscle strength) × 100]	52.6 (%)	Walking speed (m/s) 6-min walk distance (meters)	0.91 (m/s) 243.7 ± 122.2 (meters)	−0.75
Dorsch S [27]	2012	60	42/18	69 ± 11	28/32	1–6 range (years)	HHD	Strength (N)	66 ± 37 (N)	Walking speed (m/s)	0.75 ± 0.34 (m/s)	0.50
Ng SSM [28]	2025	65	35/30	66.91 ± 6.40	31/34	9.45 ± 4.99 (years)	HHD (Lafayette Instrument Corp., Lafayette, IN, USA)	Strength (kg)	9.26 ± 4.62 (kg)	Walking speed (m/s)	Usual 18.20 ± 11.57 (m/s) Maximum 14.00 ± 8.60 (m/s)	−0.48
Kwong PWH [29]	2017	105	63/42	61.0 ± 6.9	48/57	6.2 ± 4.9 (years)	HHD (Lafa yette Instrument Company, Lafayette, IN, USA)	Strength (kg)	8.5 ± 5.0 (kg)	6 MWT (m)	219.7 ± 84.4 (m)	0.41
Aguiar LT [30]	2018	44	24/20	62 ± 15	25/19	4 ± 1 (months)	Handheld microFET2^®^ dynamometer (Hoggan Scientific, LLC, Salt Lake City, UT, USA)	Isometric force (Nm/kg)	5.7 ± 2.4 (Nm/kg)	Walking speed (m/s)	Comfortable 0.8 ± 0.38 (m/s) Maximum 1.1 ± 0.54 (m/s)	0.37
Lodha N [31]	2019	21	13/8	65.04 ± 13.72	4/17	4.79 ± 4.66 (months)	Force transducer (Honeywell, Morristown, NJ, USA)	MVC (N)	143.64 ± 67.50 (N)	Walking speed (m/s)	NR	0.34
Chisholm AE [32]	2013	55	Non-dropped foot: 28/15 Dropped foot: 9/3	69.5 ± 12.2 66.8 ± 13.2	19/19 (Bilateral 5) 9/2 (Bilateral 1)	35.0 ± 11.5 (days) 57.9 ± 24.7 (days)	Load cell (Interface Inc., Scottsdale, AZ, USA)	Isometric force (MVC)	NR	Swing peak dorsiflexors	NR	−0.32
Ng SS [33]	2012	62	51/11	57.4 ± 7.8	43/19	5.2 ± 3.7(years)	load cell	Peak torque (Nm)	14.2 ± 6.7 (Nm)	Walking speed(cm/s) 6 MWT (m)	51.5 ± 26.1 (cm/s) 183.7 ± 84.4 (m)	0.79
Ng SS [34]	2013	73	60/13	57.16 ± 7.91	45/28	5.21 ± 3.63 (years)	Load cell	Peak torque (Nm)	14.67 ± 7.01 (Nm)	TUG(s)	27.41 ± 17.63 (s)	−0.67
Kowal M [35]	2020	15	7/8	57.2 ± 11	7/8	1.53 ± 0.64 (months)	Biodex System (Biodex Medical Systems Inc., Shirley, NY, USA)	Torque (Nm/kg)	0.3 (Nm/kg)	Walking speed (m/s)	0.9 ± 0.1 (m/s)	0.65
Kim CM [36]	2003	20	14/6	61.2 ± 8.4	9/11	4.0 ± 2.6 (years)	Kim-Com isokinetic dynamometer (Chattanooga Group Inc, 4717 Adams Rd, Hixson, TN, USA)	Torque (Nm)	0.15 ± 0.13 (Nm)	Walking speed (m/s) self-selected pace, maximum	Self-selected pace 0.45 ± 0.25 (m/s) Maximum 0.69 ± 0.35 (m/s)	0.33
Klein CS [37]	2010	7	5/2	55.8 ± 3.6	4/3	NR	Load cell (Omega Engineering, Stamford, CT, USA)	MVC torque (Nm)	56.7 ± 57.4 (Nm)	Walking speed (m/s)	0.83 ± 0.33 (m/s)	0.75
Johnson CA [38]	2025	39	24/15	60 ± 12	27/12	1155 ± 1096 (days)	Ankle Measuring Proprioceptive Device	Joint Position Reproduction dynamic error (°)	11.2 ± 5.6 (°)	Walking speed (m/s) 6MWT (m)	0.37 ± 0.24 (m/s) 114.4 ± 66.4 (m)	−0.28
Lee MJ [39]	2005	11	9/2	69 ± 11	8/3	43 ± 32 (months)	linear servo-motor	ROM (°)	19.5 ± 17.4 (°)	6MWT (m)	324.4 ± 173.1 (m)	0.48
Negro F [40]	2020	10	5/5	60.4 ± 13	NR	15.8 ± 10 (years)	load cell (EMG, Scottsdale, AZ, USA)	MVC (N)	106. ± 54 (N)	Walking speed (m/s)	NR	0.71
Cho KH [41]	2014	39	23/16	67.8 ± 0.9	20/19	200.1 ± 227.1 (days)	NR (non-instrumented clinical grading; MMT)	MMT	2.7 ± 1.6	Walking level (ordinal scale)	3.6 ± 1.5	0.56

NR = not reported in the original article.

**Table 2:** Summary of meta-analysis of correlations between ankle-dorsiflexor strength (or central measures) and gait performance.

				95% CI				
Group	Outcome	No. of Studies	Pooled r	Lower	Upper	Model	I^2^	τ^2^
All	Walking speed	17	0.19	−0.14	0.48	Random-effects	94.70	0.45
Strength	Walking speed	13	0.65	−0.42	0.96	Random-effects	94.52	0.40

## Data Availability

All data used in this review were derived from previously published articles cited in the manuscript. The extracted and synthesized datasets generated during the current study are available from the corresponding author (T.K.) upon reasonable request.

## Supplementary Materials

The following supporting information can be downloaded at: https://www.mdpi.com/article/10.3390/s26113598/s1. Table S1: Lists the search terms used in this review. Table S2: Lists the selected references identified through the search. Table S3: Quality assessment of the studies included in this systematic review using the Newcastle−Ottawa Scale. Each study was evaluated across three domains: Selection (S1–S3; maximum 3★), Comparability (C1–C2; maximum 2★), and Outcome (O1; maximum 1★). The selection criteria comprised the representativeness of the exposed cohort (S1), selection of the nonexposed cohort (S2), ascertainment of exposure (S3). Comparability was assessed based on controlling for age/severity (C1) and other potential confounders (C2). Outcome assessments included the method of outcome assessment (O1). Total scores were used to classify studies as high quality (5–6★), moderate quality (3–4★), or low quality (0–2★). For cross-sectional studies, items related to follow-up or outcomes not present at baseline (S4, O2, and O3) were classified as “not applicable (NA)” and were not counted as missing items in the total score.

## Abbreviations

The following abbreviations are used in this manuscript:

IMUs	inertial measurement units
sEMG	surface electromyography
PRISMA	Preferred Reporting Items for Systematic Reviews and Meta-Analyses
MeSH	Medical Subject Headings
EEG	electroencephalogram
CMC	corticomuscular coherence
MVC	maximum voluntary contraction
TUG	Timed Up and Go
HHD	hand-held dynamometer
MMT	manual muscle testing
6 MWT	6-min walk test

## References

[1] Moore S.A., Boyne P., Fulk G., Verheyden G., Fini N.A. (2022). Walk the talk: Current evidence for walking recovery after stroke, future pathways and a mission for research and clinical practice. Stroke.

[2] Li S., Francisco G.E., Zhou P. (2018). Post-stroke hemiplegic gait: New perspective and insights. Front. Physiol..

[3] Fang J., Song B., Li L., Tong L., Jiang M., Yan J. (2024). RGX Ensemble Model for Advanced Prediction of Mortality Outcomes in Stroke Patients. BME Front..

[4] Awad L.N., Palmer J.A., Pohlig R.T., Binder-Macleod S.A., Reisman D.S. (2015). Walking speed and step length asymmetry modify the energy cost of walking after stroke. Neurorehabilit. Neural Repair.

[5] Skvortsov D.V., Kaurkin S.N., Grebenkina N.V., Ivanova G.E. (2025). Typical changes in gait biomechanics in patients with subacute ischemic stroke. Diagnostics.

[6] Darak V., Karthikbabu S. (2020). Lower limb motor function and hip muscle weakness in stroke survivors and their relationship with pelvic tilt, weight-bearing asymmetry, and gait speed: A cross-sectional study. Curr. J. Neurol..

[7] Kochman M., Kasprzak M., Kielar A. (2025). The impact of proprioception impairment on gait function in stroke survivors: A comprehensive review. Front. Neurol..

[8] Jørgensen H.S., Nakayama H., Raaschou H.O., Olsen T.S. (1995). Recovery of walking function in stroke patients: The Copenhagen Stroke Study. Arch. Phys. Med. Rehabil..

[9] Peishun C., Haiwang Z., Taotao L., Hongli G., Yu M., Wanrong Z. (2021). Changes in gait characteristics of stroke patients with foot drop after the combination treatment of foot drop stimulator and moving treadmill training. Neural Plast..

[10] Gao T., Ma Z., Yang N., Zhang S., Shi H., Zhang H., Ren S., Huang H. (2024). The relationship of peak ankle dorsiflexion angle with lower extremity biomechanics during walking. J. Foot Ankle Res..

[11] Gil-Castillo J., Alnajjar F., Koutsou A., Torricelli D., Moreno J.C. (2020). Advances in neuroprosthetic management of foot drop: A review. J. Neuroeng. Rehabil..

[12] Alam M., Choudhury I.A., Bin Mamat A. (2014). Mechanism and design analysis of articulated ankle foot orthoses for drop-foot. Sci. World J..

[13] Kirdthongkham T., Justine M., Siriphorn A. (2025). Prognostic accuracy of the Stroke Rehabilitation Assessment of Movement (STREAM) scores on admission for walking independence in stroke patients at discharge and one-month follow-up. PLoS ONE.

[14] Hulleck A.A., Menoth Mohan D., Abdallah N., El Rich M., Khalaf K. (2022). Present and future of gait assessment in clinical practice: Towards the application of novel trends and technologies. Front. Med. Technol..

[15] Kumfu S., Poncumhak P. (2022). Predictive ability of the three-time stand and walk test to determine frailty and its associations with fear of falling and cognitive function in community-dwelling older adults. Ann. Geriatr. Med. Res..

[16] Steinbrink G.M., Martinez J., Swartz A.M., Strath S.J. (2024). Sit-to-stand power is a stronger predictor of gait speed than knee extension strength. J. Funct. Morphol. Kinesiol..

[17] Dobkin B.H. (2013). Wearable motion sensors to continuously measure real-world physical activities. Curr. Opin. Neurol..

[18] Xu D., Zhou H., Jie T., Zhou Z., Yuan Y., Jemni M., Quan W., Gao Z., Xiang L., Gusztav F. (2025). Data-driven deep learning for predicting ligament fatigue failure risk mechanisms. Int. J. Mech..

[19] McCain E.M., Dick T.J.M., Giest T.N., Nuckols R.W., Lewek M.D., Saul K.R., Sawicki G.S. (2019). Mechanics and energetics of post-stroke walking aided by a powered ankle exoskeleton with speed-adaptive myoelectric control. J. Neuroeng. Rehabil..

[20] Malouin F., Richards C.L. (2010). Mental practice for relearning locomotor skills. Phys. Ther..

[21] Campanini I., Disselhorst-Klug C., Rymer W.Z., Merletti R. (2020). Surface EMG in clinical assessment and neurorehabilitation: Barriers limiting its use. Front. Neurol..

[22] Page M.J., McKenzie J.E., Bossuyt P.M., Boutron I., Hoffmann T.C., Mulrow C.D., Shamseer L., Tetzlaff J.M., Akl E.A., Brennan S.E. (2021). The PRISMA 2020 statement: An updated guideline for reporting systematic reviews. BMJ.

[23] Wells G.A., Shea B., O’Connell D., Peterson J., Welch V., Losos M., Tugwell P. (2014). The Newcastle-Ottawa Scale (NOS) for Assessing the Quality of Nonrandomised Studies in Meta-Analyses.

[24] Mentiplay B.F., Williams G., Tan D., Adair B., Pua Y.H., Bok C.W., Bower K.J., Cole M.H., Ng Y.S., Lim L.S. (2019). Gait velocity and joint power generation after stroke: Contribution of strength and balance. Am. J. Phys. Med. Rehabil..

[25] Chan P.P., Si Tou J.I., Tse M.M., Ng S.S. (2017). Reliability and validity of the timed up and Go test with a motor task in people with chronic stroke. Arch. Phys. Med. Rehabil..

[26] Ozgozen S., Guzel R., Basaran S., Coskun Benlidayi I. (2020). Residual deficits of knee flexors and plantar flexors predict normalized walking performance in patients with poststroke hemiplegia. J. Stroke Cerebrovasc. Dis..

[27] Dorsch S., Ada L., Canning C.G., Al-Zharani M., Dean C. (2012). The strength of the ankle dorsiflexors has a significant contribution to walking speed in people who can walk independently after stroke: An observational study. Arch. Phys. Med. Rehabil..

[28] Ng S.S.M., Chen P., Usman J.S., Chan P.H., Chau K.K., Lee S.H., Liang C.Y., Man T.K., Hsu C.L., Li K.J.J. (2025). Relationship of lower-limb muscle properties with motor control, walking and balance in stroke survivors. Sci. Rep..

[29] Kwong P.W.H., Ng S.S.M., Chung R.C.K., Ng G.Y.F. (2017). A structural equation model of the relationship between muscle strength, balance performance, walking endurance and community integration in stroke survivors. PLoS ONE.

[30] Aguiar L.T., Camargo L.B.A., Estarlino L.D., Teixeira-Salmela L.F., Faria C.D.C.M. (2018). Strength of the lower limb and trunk muscles is associated with gait speed in individuals with sub-acute stroke: A cross-sectional study. Braz. J. Phys. Ther..

[31] Lodha N., Patel P., Casamento-Moran A., Hays E., Poisson S.N., Christou E.A. (2019). Strength or motor control: What matters in high-functioning stroke?. Front. Neurol..

[32] Chisholm A.E., Perry S.D., McIlroy W.E. (2013). Correlations between ankle-foot impairments and dropped foot gait deviations among stroke survivors. Clin. Biomech..

[33] Ng S.S., Hui-Chan C.W. (2012). Contribution of ankle dorsiflexor strength to walking endurance in people with spastic hemiplegia after stroke. Arch. Phys. Med. Rehabil..

[34] Ng S.S.M., Hui-Chan C.W.Y. (2013). Ankle dorsiflexor, not plantarflexor strength, predicts the functional mobility of people with spastic hemiplegia. J. Rehabil. Med..

[35] Kowal M., Kołcz A., Dymarek R., Paprocka-Borowicz M., Gnus J. (2020). Muscle torque production and kinematic properties in post-stroke patients: A pilot cross-sectional study. Acta Bioeng. Biomech..

[36] Kim C.M., Eng J.J. (2003). The relationship of lower-extremity muscle torque to locomotor performance in people with stroke. Phys. Ther..

[37] Klein C.S., Brooks D., Richardson D., McIlroy W.E., Bayley M.T. (2010). Voluntary activation failure contributes more to plantar flexor weakness than antagonist coactivation and muscle atrophy in chronic stroke survivors. J. Appl. Physiol..

[38] Johnson C.A., Biswas P., Tapia R., See J., Dodakian L., Chan V., Wang P.T., Nenadic Z., Do A.H., Reinkensmeyer D.J. (2025). The weak relationship between ankle proprioception and gait speed after stroke: A robotic assessment study. Neurorehabilit. Neural Repair.

[39] Lee M.J., Kilbreath S.L., Refshauge K.M. (2005). Movement detection at the ankle following stroke is poor. Aust. J. Physiother..

[40] Negro F., Bathon K.E., Nguyen J.N., Bannon C.G., Orizio C., Hunter S.K., Hyngstrom A.S. (2020). Impaired firing behavior of individually tracked paretic motor units during fatiguing contractions of the dorsiflexors and functional implications post stroke. Front. Neurol..

[41] Cho K.H., Lee J.Y., Lee K.J., Kang E.K. (2014). Factors related to gait function in post-stroke patients. J. Phys. Ther. Sci..

[42] Kwakkel G., Kollen B., Twisk J. (2006). Impact of time on improvement of outcome after stroke. Stroke.

[43] Lins C., Oliveira A.R.B., Chagas M.S.S., Ribeiro F., Cliquet A., Pagnano R.G. (2025). Assessment of Isometric and Isokinetic Ankle Strength Measures: A Pilot Study. Acta Ortop. Bras..

[44] Yasacı Z., Argut S.K., Celik D. (2024). Reliability of a new stabilization device for measurement of muscle strength using a hand-held dynamometer. Muscle Nerve.

[45] Sahu P.K., Goodstadt N., Ramakrishnan A., Silfies S.P. (2024). Test-retest reliability and concurrent validity of knee extensor strength measured by a novel device incorporated into a weight stack machine vs. handheld and isokinetic dynamometry. PLoS ONE.

[46] Picot B., Maricot A., Fourchet F., Gokeler A., Tassignon B., Lopes R., Hardy A. (2025). Targeting visual-sensory and cognitive impairments following lateral ankle sprains: A practical framework for functional assessment across the return-to-sport continuum-Part 1. Sensory reweighting and cognitive impairments: What are we really talking about and why clinicians should consider central alterations in return to sport criteria. Front. Sports Act. Living.

[47] Jastifer J.R. (2025). Contemporary Review: Proprioception in Ankle Stability. Foot Ankle Orthop..

[48] Kwakkel G., Kollen B.J., van der Grond J., Prevo A.J.H. (2003). Probability of regaining dexterity in the flaccid upper limb: Impact of severity of paresis and time since onset in acute stroke. Stroke.

[49] Ogborn D.I., Bellemare A., Bruinooge B., Brown H., McRae S., Leiter J. (2021). Comparison of Common Methodologies for the Determination of Knee Flexor Muscle Strength. Int. J. Sports Phys. Ther..

[50] Sul J.H., Piyathilaka L., Moratuwage D., Dunu Arachchige S., Jayawardena A., Kahandawa G., Preethichandra D.M.G. (2025). Electromyography Signal Acquisition, Filtering, and Data Analysis for Exoskeleton Development. Sensors.

[51] Fang C., He B., Wang Y., Cao J., Gao S. (2020). EMG-Centered Multisensory Based Technologies for Pattern Recognition in Rehabilitation: State of the Art and Challenges. Biosensors.

[52] Hohsoh N., Sanghan T., Chong D.Y.R., Stojanovic G., Chatpun S. (2024). Comparative electromyography analysis of subphase gait disorder in chronic stroke survivors. PeerJ.

[53] Srivastava S., Patten C., Kautz S.A. (2019). Altered muscle activation patterns (AMAP): An analytical tool to compare muscle activity patterns of hemiparetic gait with a normative profile. J. Neuroeng. Rehabil..

[54] Maffiuletti N.A., Aagaard P., Blazevich A.J., Folland J., Tillin N., Duchateau J. (2016). Rate of force development: Physiological and methodological considerations. Eur. J. Appl. Physiol..

[55] Winter D.A., Yack H.J. (1987). EMG profiles during normal human walking: Stride-to-stride and inter-subject variability. Electroencephalogr. Clin. Neurophysiol..

[56] Hillier S., Immink M., Thewlis D. (2015). Assessing proprioception: A systematic review of possibilities. Neurorehabilit. Neural Repair.

[57] Aman J.E., Elangovan N., Yeh I.L., Konczak J. (2015). The effectiveness of proprioceptive training for improving motor function: A systematic review. Front. Hum. Neurosci..

[58] Fulk G.D., Echternach J.L. (2008). Test-retest reliability and minimal detectable change of gait speed in individuals undergoing rehabilitation after stroke. J. Neurol. Phys. Ther..

[59] Flansbjer U.B., Holmbäck A.M., Downham D., Patten C., Lexell J. (2005). Reliability of gait performance tests in men and women with hemiparesis after stroke. J. Rehabil. Med..

[60] Bohannon R.W. (2007). Muscle strength and muscle training after stroke. J. Rehabil. Med..

[61] Ada L., Dorsch S., Canning C.G. (2006). Strengthening interventions increase strength and improve activity after stroke: A systematic review. Aust. J. Physiother..

