# Supplementary material for: Measurement Technologies for Ankle-Dorsiflexion Function After Stroke: A Systematic Review and Meta-Analysis of Sensing Approaches and Their Relationships with Gait Performance
Source: Sensors (Basel). 2026 Jun 5;26(11):3598. doi: 10.3390/s26113598 (PMC13259174; doi:10.3390/s26113598)
Supplement: Supplementary file 1 [file sensors-26-03598-s001.zip › Supplementary Table S1.pdf]

Supplementary Table S1. List of Search Strategy

Database	Search Strategy
PubMed	"Stroke" OR "Hemiplegia" OR "Cerebrovascular accident") AND ("Ankle" OR "Dorsiflexion" OR "Tibialis anterior") AND ("Strength" OR "Muscle force" OR "Torque" OR "Dynamometer" OR "Manual muscle test" OR maximum voluntary contraction ("MVC") OR "electroencephalogram (EEG)" OR "Bereitschaftspotential" OR "Readiness potential" OR "Motor-evoked potential" OR "Corticospinal excitability" OR "Voluntary activation") AND ("Gait speed" OR "Walking speed" OR "Walking capacity" OR "6 minute walk" OR "Timed Up and Go" (TUG) OR "Correlation" OR "Relationship" OR "Association" OR "Predict*"
IEEE Xplore	"Stroke" OR "Hemiplegia" OR "Cerebrovascular accident") AND ("Ankle" OR "Dorsiflexion" OR "Tibialis anterior") AND ("Strength" OR "Muscle force" OR "Torque" OR "Dynamometer" OR "Manual muscle test" OR "MVC" OR "EEG" OR "Bereitschaftspotential" OR "Readiness potential" OR "Motor evoked potential" OR "Corticospinal excitability" OR "Voluntary activation") AND ("Gait speed" OR "Walking speed" OR "Walking capacity" OR "6 minute walk" OR "Timed Up and Go" OR "Correlation" OR "Relationship" OR "Association" OR "Predict*"
Cochrane Library	"Stroke" OR "Hemiplegia" OR "Cerebrovascular accident") AND ("Ankle" OR "Dorsiflexion" OR "Tibialis anterior") AND ("Strength" OR "Muscle force" OR "Torque" OR "Dynamometer" OR "Manual muscle test" OR "MVC" OR "EEG" OR "Bereitschaftspotential" OR "Readiness potential" OR "Motor evoked potential" OR "Corticospinal excitability" OR "Voluntary activation") AND ("Gait speed" OR "Walking speed" OR "Walking capacity" OR "6 minute walk" OR "Timed Up and Go" OR "Correlation" OR "Relationship" OR "Association" OR "Predict*"
