# Supplementary material for: Measurement Technologies for Ankle-Dorsiflexion Function After Stroke: A Systematic Review and Meta-Analysis of Sensing Approaches and Their Relationships with Gait Performance
Source: Sensors (Basel). 2026 Jun 5;26(11):3598. doi: 10.3390/s26113598 (PMC13259174; doi:10.3390/s26113598)
Supplement: Supplementary file 1 [file sensors-26-03598-s001.zip › Supplementary Table S2.pdf]

Supplementary Table S2. List of Articles

title	authors	journal
Gait Velocity and Joint Power Generation After Stroke: Contribution of Strength and Balance	Mentiplay BF, Williams G, Tan D, Adair B, Pua YH, Bok CW, Bower KJ, Cole MH, Ng YS, Lim LS, Clark RA. [24]	Am J Phys Med Rehabil. 2019 Oct;98(10):841-849. doi: 10.1097/PHM.0000000000001122.
Reliability and Validity of the Timed Up and Go Test With a Motor Task in People With Chronic Stroke	Chan PP, Si Tou JI, Tse MM, Ng SS. [25]	Arch Phys Med Rehabil. 2017 Nov;98(11):2213-2220. doi: 10.1016/j.apmr.2017.03.008. Epub 2017 Apr 7.

Residual Deficits of Knee Flexors and Plantar Flexors Predict Normalized Walking Performance in Patients with Poststroke Hemiplegia	Ozgozen S, Guzel R, Basaran S, Coskun Benlidayi I. [26]	J Stroke Cerebrovasc Dis. 2020 Apr;29(4):104658. doi: 10.1016/j.jstrokecerebrovasdis.2020.104658. Epub 2020 Feb 7.
The strength of the ankle dorsiflexors has a significant contribution to walking speed in people who can walk independently after stroke: an observational study	Dorsch S, Ada L, Canning CG, Al-Zharani M, Dean C. [27]	Arch Phys Med Rehabil. 2012 Jun;93(6):1072-6. doi: 10.1016/j.apmr.2012.01.005. Epub 2012 Mar 29.
Relationship of lower-limb muscle properties with motor control, walking and balance in stroke survivors	Ng SSM, Chen P, Usman JS, Chan PH, Chau KK, Lee SH,	Sci Rep. 2025 Dec 26;16(1):2563. doi: 10.1038/s41598-025-32527-4.

	Liang CY, Man TK, Hsu CL, Li KJJ, Tse MMY. [28]	
A structural equation model of the relationship between muscle strength, balance performance, walking endurance and community integration in stroke survivors	Kwong PWH, Ng SSM, Chung RCK, Ng GYF. [29]	PLoS One. 2017 Oct 19;12(10):e0185807. doi: 10.1371/journal.pone.0185807. eCollection 2017.
Strength of the lower limb and trunk muscles is associated with gait speed in individuals with sub-acute stroke: a cross-sectional study	Aguiar LT, Camargo LBA, Estarlino LD, Teixeira-Salmela LF, Faria CDCM. [30]	Braz J Phys Ther. 2018 Nov-Dec;22(6):459-466. doi: 10.1016/j.bjpt.2018.03.001. Epub 2018 Mar 15.

Strength or Motor Control: What Matters in High-Functioning Stroke?	Lodha N, Patel P, Casamento-Moran A, Hays E, Poisson SN, Christou EA. [31]	Front Neurol. 2019 Jan 9;9:1160. doi: 10.3389/fneur.2018.01160. eCollection 2018.
Correlations between ankle-foot impairments and dropped foot gait deviations among stroke survivors	Chisholm AE, Perry SD, McIlroy WE. [32]	Clin Biomech (Bristol). 2013 Nov-Dec;28(9-10):1049-54. doi: 10.1016/j.clinbiomech.2013.09.007. Epub 2013 Sep 19.
Contribution of ankle dorsiflexor strength to walking endurance in people with spastic hemiplegia after stroke	Ng SS, Hui-Chan CW. [33]	Arch Phys Med Rehabil. 2012 Jun;93(6):1046-51. doi: 10.1016/j.apmr.2011.12.016. Epub 2012 Mar 20.

Ankle dorsiflexor, not plantarflexor strength, predicts the functional mobility of people with spastic hemiplegia	Ng SS, Hui-Chan CW. [34]	J Rehabil Med. 2013 Jun;45(6):541-5. doi: 10.2340/16501977-1154.
Muscle torque production and kinematic properties in post-stroke patients: a pilot cross-sectional study	Kowal M, Kołcz A, Dymarek R, Paprocka-Borowicz M, Gnus J. [35]	Acta Bioeng Biomech. 2020;22(1):11-20.
The relationship of lower-extremity muscle torque to locomotor performance in people with stroke	Kim CM, Eng JJ. [36]	Phys Ther. 2003 Jan;83(1):49-57.
Voluntary activation failure contributes more to plantar flexor weakness than antagonist	Klein CS, Brooks D, Richardson D, McIlroy WE, Bayley MT. [37]	J Appl Physiol (1985). 2010 Nov;109(5):1337-46. doi:

coactivation and muscle atrophy in chronic stroke survivors		10.1152/japplphysiol.00804.2009. Epub 2010 Aug 19.
The Weak Relationship Between Ankle Proprioception and Gait Speed After Stroke: A Robotic Assessment Study	Johnson CA, Biswas P, Tapia R, See J, Dodakian L, Chan V, Wang PT, Nenadic Z, Do AH, Reinkensmeyer DJ. [38]	Neurorehabil Neural Repair. 2025 Dec;39(12):1031-1045. doi: 10.1177/15459683251369497. Epub 2025 Sep 16.
Movement detection at the ankle following stroke is poor	Lee MJ, Kilbreath SL, Refshauge KM. [39]	Aust J Physiother. 2005;51(1):19-24. doi: 10.1016/s0004-9514(05)70049-0.
Impaired Firing Behavior of Individually Tracked Paretic Motor Units During Fatiguing	Negro F, Bathon KE, Nguyen JN, Bannon CG, Orizio C, Hunter SK, Hyngstrom AS. [40]	Front Neurol. 2020 Oct 29;11:540893. doi: 10.3389/fneur.2020.540893. eCollection 2020.

Contractions of the Dorsiflexors and Functional Implications Post Stroke		
Factors Related to Gait Function in Post-stroke Patients	Cho KH, Lee JY, Lee KJ, Kang EK. [41]	J Phys Ther Sci. 2014 Dec;26(12):1941-4. doi: 10.1589/jpts.26.1941. Epub 2014 Dec 25.
