# Supplementary material for: Measurement Technologies for Ankle-Dorsiflexion Function After Stroke: A Systematic Review and Meta-Analysis of Sensing Approaches and Their Relationships with Gait Performance
Source: Sensors (Basel). 2026 Jun 5;26(11):3598. doi: 10.3390/s26113598 (PMC13259174; doi:10.3390/s26113598)
Supplement: Supplementary file 1 [file sensors-26-03598-s001.zip › Supplementary Table S3.pdf]

Supplementary Table S3. Quality Assessment of Included Studies Using Newcastle-Ottawa Scale

Study	Selection (max 4★)				Comparability (max 2★)		Outcome (max 3★)			Total	Rating
	S1	S2	S3	S4	C1	C2	O1	O2	O3		
Mentiplay BF et al. 2019[24]	★	★	★	NA	★	★	★	NA	NA	6	High
Lodha N et al. 2019[31]	★	★	★	NA	★	★	★	NA	NA	6	High
Chan PP et al. 2017[25]	★	★	★	NA	★	★	★	NA	NA	6	High
Kowal M et al. 2020[35]	★	★	★	NA	★	–	★	NA	NA	5	High
Chisholm AE et al. 2013[32]	★	★	★	NA	★	–	★	NA	NA	5	High
Aguiar LT et al. 2018[30]	★	★	★	NA	★	–	★	NA	NA	5	High
Ozgozen S et al. [26]	★	★	★	NA	★	–	★	NA	NA	5	High
Dorsch S et al. 2012[27]	★	★	★	NA	★	–	★	NA	NA	5	High
Ng SS & Hui-Chan CW. 2013[34]	★	★	★	NA	★	★	★	NA	NA	6	High
Klein CS et al. 2010[37]	★	★	★	NA	★	–	★	NA	NA	5	High
Johnson CA et al. 2025[38]	★	★	★	NA	★	★	★	NA	NA	6	High
Ng SSM et al. 2025[28]	★	★	★	NA	★	★	★	NA	NA	6	High
Ng SS & Hui-Chan CW. 2012 [33]	★	★	★	NA	★	★	★	NA	NA	6	High

Study	Selection (max 4★)				Comparability (max 2★)		Outcome (max 3★)			Total	Rating
	S1	S2	S3	S4	C1	C2	O1	O2	O3		
Kim CM & Eng JJ. 2003[36]	★	★	★	NA	★	–	★	NA	NA	5	High
Cho KH et al. 2014[41]	★	★	★	NA	★	–	★	NA	NA	5	High
Negro F et al. 2020[40]	★	★	★	NA	★	–	★	NA	NA	5	High
Kwong PWH et al. 2017[29]	★	★	★	NA	★	★	★	NA	NA	6	High
Lee MJ et al. 2005[39]	★	★	★	NA	★	★	★	NA	NA	6	High

Selection criteria: S1 = Representativeness of exposed cohort; S2 = Selection of non-exposed cohort; S3 = Ascertainment of exposure; S4 = Demonstration outcome not present at start.

Comparability: C1 = Control for age/severity; C2 = Control for other factors.

Outcome: O1 = Assessment of outcome; O2 = Adequate follow-up length; O3 = Adequacy of follow-up.

Quality ratings (adapted for cross-sectional designs, maximum 6★): High = 5–6★; Moderate = 3–4★; Low = 0–2★.

Items S4, O2, and O3 were considered not applicable for cross-sectional designs and were therefore coded as “NA” and not penalized when calculating total scores.
